# The simultaneous ex vivo detection of low-frequency antigen-specific CD4+ and CD8+ T-cell responses using overlapping peptide pools

**DOI:** 10.1007/s00262-012-1251-3

**Published:** 2012-04-11

**Authors:** Satwinder Kaur Singh, Maaike Meyering, Tamara H. Ramwadhdoebe, Linda F. M. Stynenbosch, Anke Redeker, Peter J. K. Kuppen, Cornelis J. M. Melief, Marij J. P. Welters, Sjoerd H. van der Burg

**Affiliations:** 1 Department of Clinical Oncology, Building 1, K1-P, Leiden University Medical Center, PO box 9600, 2300 RC Leiden, The Netherlands; 2 Department of Immunohematology and Blood Transfusion, Leiden University Medical Center, Leiden, The Netherlands; 3 Department of Surgery, Leiden University Medical Center, Leiden, The Netherlands

**Keywords:** Intracellular cytokine staining, Flow cytometry, Monocytes, Immunomonitoring, T cells

## Abstract

The ability to measure antigen-specific T cells at the single-cell level by intracellular cytokine staining (ICS) is a promising immunomonitoring tool and is extensively applied in the evaluation of immunotherapy of cancer. The protocols used to detect antigen-specific CD8+ T-cell responses generally work for the detection of antigen-specific T cells in samples that have undergone at least one round of in vitro pre-stimulation. Application of a common protocol but now using long peptides as antigens was not suitable to simultaneously detect antigen-specific CD8+ and CD4+ T cells directly ex vivo in cryopreserved samples. CD8 T-cell reactivity to monocytes pulsed with long peptides as antigens ranged between 5 and 25 % of that observed against monocytes pulsed with a direct HLA class I fitting minimal CTL peptide epitope. Therefore, we adapted our ICS protocol and show that the use of tenfold higher concentration of long peptides to load APC, the use of IFN-α and poly(I:C) to promote antigen processing and improve T-cell stimulation, does allow for the ex vivo detection of low-frequency antigen-specific CD8+ and CD4+ T cells in an HLA-independent setting. While most of the improvements were related to increasing the ability to measure CD8+ T-cell reactivity following stimulation with long peptides to at least 50 % of the response detected when using a minimal peptide epitope, the final analysis of blood samples from vaccinated patients successfully showed that the adapted ICS protocol also increases the ability to ex vivo detect low-frequency p53-specific CD4+ T-cell responses in cryopreserved PBMC samples.

**Electronic supplementary material:**

The online version of this article (doi:10.1007/s00262-012-1251-3) contains supplementary material, which is available to authorized users.

## Introduction

A large body of data generated by mechanistic studies in animal models showed that both tumor-specific CD4+ T-helper type (Th) 1 and cytotoxic CD8+ T cells (CTL) play a major role in controlling tumor growth [1–3]. Cohort studies indicating an increased incidence of cancer in immune-suppressed patients or showing that the presence of memory CD4+ Th1 and CTL in tumors is predictive for a beneficial clinical outcome as well as clinical trials in which patients display clinical benefit after adoptive transfer of tumor-specific T cells or after therapeutic vaccination sustain these conclusions [4]. Recent data from immunotherapy studies suggest that the expansion of antigen-specific infused T cells [5, 6] or the magnitude, type and breadth of the vaccine-induced T-cell reaction [7–11] may correlate with success or failure to respond to treatment and reinforces the notion that the ability to type and enumerate T-cell reactivity within clinical samples is an important asset in the development of new treatments for cancer. Evidently, the number and type of assays that can be used are determined by logistics. In most cases, a number of only relatively small (50 mL) blood samples are taken because patients have no problems consenting to this and because it is easy to isolate peripheral blood mononuclear cells (PBMC) and to store them in liquid nitrogen for later studies. Different techniques have been developed to measure and enumerate the T-cell response to as many as possible epitopes within one sample directly ex vivo. One such method to gain information about T-cell reactivity at feasible extent constitutes the stimulation of PBMC with overlapping peptide pools of defined antigens. This allows for simultaneous testing of functional reactivity of both CD4+ and CD8+ T cells after vaccination or during viral infection [12, 13], both by IFN-γ ELISPOT assay and by the flow cytometry-based intracellular cytokine staining (ICS), with the latter being less equipped to measure low-level responses [14]. In addition, pools containing peptides that are 5–6 amino acids longer than the exact HLA class I-restricted T-cell epitopes resulted in the detection <80 % of the frequencies found than when T cells were stimulated with the exact HLA-fitting peptide epitope, although they were appropriate for the stimulation of CD4+ T cells [15]. We have used single or pooled 30-mer peptides for the detection of CD4+ and CD8+ T-cell responses against influenza matrix protein 1 (M1), following one round of enrichment and expansion [16], under the premise that this would also allow us to screen T-cell reactivity against a high number of pooled antigens if patient samples are limiting. While this allowed us to stimulate both CD4 and CD8 T cells during 10-day cultures as well as to measure CD4+ T-cell reactivity, we needed to return to large pools of 10-mer peptides to appropriately measure CD8+ T-cell reactivity against HPV antigens [10, 11], as it was difficult to separate populations of responding CD8+ T cells from the background.

In order to optimize the simultaneous detection of both IFN-γ-producing CD8+ and CD4+ T cells using pools of long peptides directly ex vivo, we exploited the T-cell response to influenza M1, as these responses are present in the majority of humans at similar frequencies that are expected to arise after vaccination with cancer vaccines. Based on our previous IFN-γ-ELISPOT analyses, influenza M1-specific CD4+ T-cell responses and the HLA-A*0201-restricted influenza M1 CD8+ T-cell epitope are present in frequencies between 1/10,000 and 1/1,000 [17–20], which is about the frequency of tumor-specific T cells after vaccination [8, 11].

## Materials and methods

The authors acknowledge the concept of the Minimal Information About T cell Assays (MIATA) framework, which was recently published [21, 22]. Therefore, detailed information is provided as structured in the proposed 5 modules by MIATA: the sample, the assay, the data acquisition, the data analysis and the laboratory environment in which the human T-cell assays were performed.

### Media and reagents

IMDM (Lonza, Verviers, Belgium), supplemented with 100 U/mL penicillin/100 μL/mL streptomycin (Invitrogen, Grand Island, NY, USA), 2 mM l-glutamine (Cambrex, East Rutherford, NJ, USA) and human AB serum (Greiner, Alphen aan den Rijn, the Netherlands), assigned as complete IMDM, or X-Vivo 15 medium (Lonza) were used as indicated. The following peptides were used in this study: CMVpp65 495-503 (CMV **S**hort **P**eptide or CMV **SP**), CMVpp65 483-512 (CMV **S**ingle **L**ong **P**eptide or CMV **SLP**), Influenza M1 58-66 (SP) and peptides spanning the whole M1 protein derived from influenza, consisting of 16 peptides with a length of 30 amino acids and an overlap of 15 amino acids (C-terminal peptide with an overlap of 18 amino acids; SLP, **L**ong **P**eptide **P**ool 1, 2, 3, 4 or **LPP**1, 2, 3, 4, each pool consist of 4 long peptides), were synthesized with >95 % purity [23] and dissolved in DMSO at the concentration of 50 mg/mL and then further diluted to a concentration of 1 mg/mL in phosphate buffered saline (PBS) and stored at −20 °C. The cytokines used in this study were GM-CSF (800 IU/mL; Immunotools, Friesoythe, Germany) and interferon alpha (Roferon-A, which is IFN-α2a). Memory Response Mix (MRM; stock 4×), consisting of tetanus toxoid (0.06 LF/mL; National Institute of Public Health and the Environment, Bilthoven, The Netherlands), mycobacterium tuberculosis sonicate (0.4 μg/mL; Royal Tropical Institute, Amsterdam, The Netherlands) and Candida Albicans (0.0012 %; HAL Allergenen Lab, Haarlem, The Netherlands).

### The sample

PBMC used in this study were derived from anonymous HLA-A*0201 healthy blood bank donors (Sanquin, The Netherlands) and from 3 patients vaccinated with a p53 vaccine in the LUMC, after informed consent. PBMC were isolated within 24 h after blood drawl by Ficoll density gradient centrifugation and cryopreserved in 90 % Fetal Calf Serum (FCS; PAA laboratories, Pasching, Austria) and 10 % DMSO (Sigma, St Louis, MO, USA). Cells were stored in the vapor phase of the liquid nitrogen vessel until further use. The handling and storage of the blood samples were performed according to the standard operating procedure (SOP) of the department of Clinical Oncology, section Experimental Cancer Immunology and Therapy at the Leiden University Medical Center by well-trained personnel.

### IFN-γ-ELISPOT

#### T-helper ELISPOT

The cryopreserved PBMC were thawed and subjected to the T-helper ELISPOT assay, both according to SOPs and as described previously [8, 10, 11, 19, 20]. Briefly, PBMC were seeded at a density of 2 × 10^6 ^cells per well in a 24-wells plate (Costar) in 1 mL of complete IMDM in the presence or absence of 5 μg/mL of indicated influenza M1-derived 30-mers peptides combined in pools (LPP1, 2, 3 and 4). As a positive control, PBMC were cultured in the presence of MRM. After 4 days of stimulation in the incubator (37 °C, 5 % CO_2_, 92 % RH), PBMC were harvested, washed, resuspended in complete IMDM (however, the 10 % human AB serum was exchanged for 10 % FCS) and seeded in four replicate wells at a density of 10^5^ cells per well in a Multiscreen 96-well plate (MAHAS45, Millipore, Billarica, MA, USA) coated with the IFN-γ-catching antibody (Mab-1-D1K, Mabtech, Nacka Strand, Sweden). Further antibody incubations and development of the ELISPOT was done according to the manufacturer’s instructions (Mabtech). Spots were counted with a fully automated computer-assisted video-imaging analysis system (BioSys 5000). Specific spots were calculated by subtracting the mean number of spots + 2 × SD of the medium only control from the mean number of spots in experimental wells. Antigen-specific T-cell frequencies were considered to be positive when specific T-cell frequencies were ≥1 of 10,000 [19].

#### CTL ELISPOT

To determine the frequency of antigen-specific CD8+ T cells, a CTL ELISPOT was conducted as according to our SOP, which is also provided on the CIMT website (http://www.cimt.eu/dl/sop_elispot.pdf). Briefly, after thawing, the PBMC were rested overnight in complete IMDM in the incubator (37 °C, 5 % CO2, 92 % RH). The following day, the cells were washed, resuspended in complete IMDM (however, the 10 % human AB serum was exchanged for 10 % FCS) and seeded at a density of 5 × 10^5 ^cells per well in the with IFN-γ-catching antibody-coated ELISPOT plate (Millipore), in the presence or absence of 1 μg/mL of indicated influenza M1 or CMV short peptide (SP). As a positive control, PBMC were stimulated on the plate with PHA. The staining and the analysis were similar as described above for the T-helper ELISPOT.

### Antigen-specific T-cell stimulation and ICS

The T-cell stimulation and staining as it was finally performed are described in the SOP shown as online resource 1. A positive response was defined as a frequency of antigen-specific T cells in the test sample, which was at least twice that of the non-stimulated PBMC (negative control). Although the definition of clearly clustered population is subjective, this was taken along in the interpretation of whether a response was found to be positive or not.

### Statistical methods

In all experiments, the mean of the triplets of IFN-γ+ T cells (CD8+ or CD4+) for each donor and antigen and the coefficient of variation (CV) was calculated. To compare the influence of IFN-α on the percentage of IFN-γ+ CD8+ cells against SLP, a paired students *t* test was used.

### Laboratory environment

The laboratory of the Clinical Oncology, section Experimental Cancer Immunology and Therapy at the Leiden University Medical Center, is a research laboratory where the assays are performed according to SOPs, including the predefined criteria for positive responses, by well-trained personnel.

## Results

### High-, intermediate- and low-frequency IFN-γ-producing CD8 T cells are detectable by intracellular cytokine staining and flow cytometry analysis when exact CTL-epitope peptides are used

**Fig. 1:**
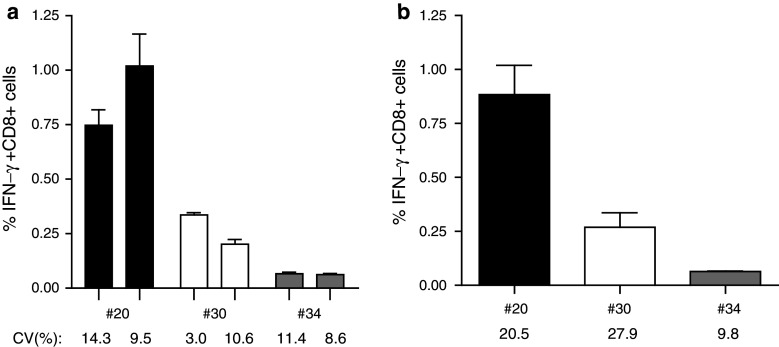
Influenza M1-derived SP (CTL-epitope) restricted CD8 T-cell responses. Different donors were tested by ICS, out of which three donors #20, 30 and 34 are depicted here. Responses shown are directed against the Influenza M1-derived short peptide (SP). **a** Two independent experiments for each donor and the intra-assay variation between the triplicates in each experiment per donor are depicted [medium background ± SD, #20: (0.023 ± 0.007), (0.005 ± 0.001); #30: (0.007 ± 0.003), (0.014 ± 0.012); #34: (0.015 ± 0.007), (0.017 ± 0.005)]. **b** Average of two independent measurements for each donor is represented with respective inter-assay variation (coefficient of variation; %CV) below 30 %

We used influenza M1 as a model antigen, as this antigen is known to activate broad CD4+ and CD8+ T-cell responses at varying frequencies ranging from low to high. First, PBMC from 16 HLA-A*0201 donors were screened for the presence of influenza M1-specific T-cell responses by IFN-γ-ELISPOT (both T-helper and CTL ELISPOT) [11, 19, 24], 15 of whom showed a response in either the T-helper and/or the CTL ELISPOT (not shown). Subsequently, positive PBMC samples were used to show the validity of our ICS protocol for measuring CD8+ T-cell responses. For that, plastic adherent monocytes were used as APC, which were activated with GM-CSF and pulsed with the exact known influenza M1-derived HLA-A*0201-restricted GILGFVFTL peptide (referred to as short peptide or SP). The non-adherent fraction of PBMC was used as responder cells, so that only one single vial of PBMC was needed for the entire experiment. Each test was performed in triplicate from the start. Figure 1 depicts the percentage of IFN-γ-producing CD8+ T cells detected (including the intra- and inter-assay variation) and shows that the magnitude of the CD8+ T-cell response against this influenza M1-derived CTL peptide varies between three different donors ranging from about 0.06–1 %. The gating strategy is shown in online resource 2. Notably, the variation between the triplicates (intra-assay) was low with covariance values ranging between 3 and 15 %. In addition, when the measurements of the influenza M1-specific IFN-γ+ CD8+ responses were repeated in independent experiments, the variation remained low with inter-assay variation well below 30 % (Fig. 1b). In conclusion, the ICS protocol used was robust enough to detect low-, intermediate- and high-frequency influenza-specific CD8+ T-cell responses allowing us to optimize the assay for the detection of CD8+ T-cell reactivity following stimulation with a single 30-mer long peptide (SLP) containing this CD8+ T-cell epitope or a pool of 16 overlapping (by 15 amino acids) 30-mers representing the influenza M1 protein including that one long peptide (LPP).

### The use of IFN-α increases the detection of CD8+ T-cell reactivity against LPP in cultures of stimulated PBMC

**Fig. 2:**
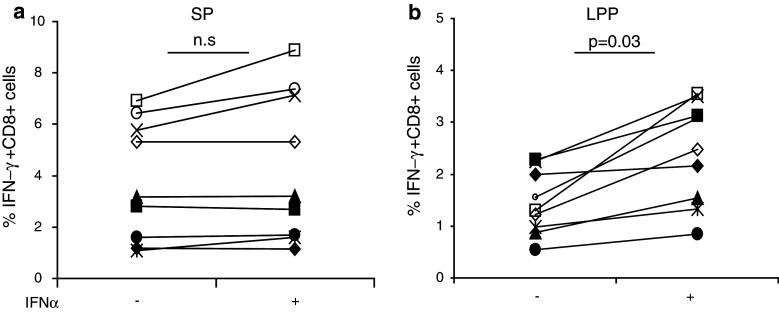
IFN-α facilitates better presentation of LPP to CD8+ T cells. PBMC from 9 different HLA-A*0201+ donors (indicated by *different symbols*) were stimulated for 12 days with the influenza M1 long peptide pools (LPP), containing 16 peptides, and then tested for reactivity by stimulating the cultured cells with monocytes pulsed with either 5 μg/mL SP (**a**) or LPP (**b**). Shown are the frequencies of IFN-γ+ CD8+ T cells against SP and LPP in the absence or presence of IFN-α. IFN-α marginally effects the SP response, but significantly boost the LPP responses (*p* = 0.03, paired *t* test)

Previously, we were able to detect influenza-specific CD8+ T-cell responses in PBMC that were stimulated with the LPP of influenza M1 and subsequently enriched and expanded [16]. Therefore, PBMC from HLA-A*0201+ donors were cultured accordingly for 12 days and then stimulated with monocytes pulsed for 24 h with either the SP or the LPP in the presence of GM-CSF. Comparison of the data obtained after SP or LPP stimulation revealed the inferior capacity of LPP-pulsed monocytes to stimulate influenza-specific CD8+ T cells to produce IFN-γ (not shown). Reports showing that IFN-α may drive cross-priming [25–27], a prerequisite for the processing and presentation of long peptides in HLA class I, made us decide to add IFN-α to the peptide-pulsed APC. Therefore, PBMC cultures were set up, and the influenza-specific CD8+ T-cell response was tested using monocytes treated with or without IFN-α. On average, the reactivity measured against the LPP was 50–60 % of the response found with SP. Whereas the response of T cells when stimulated with SP was not influenced by IFN-α (Fig. 2a), their reactivity significantly increased (*p* = 0.03, paired *t* test) when LPP-pulsed APC were treated with IFN-α (Fig. 2b). Occasionally, we found that the response measured with LPP exceeded that of reactivity found after stimulation with SP. This may be explained by additional CD8+ T-cell epitopes present in the pool of peptides.

### The detection of CD8+ T-cell responses ex vivo requires APC pulsed with much higher concentrations of single long peptides than short peptides and may be improved by the use of poly(I:C)

**Fig. 3:**
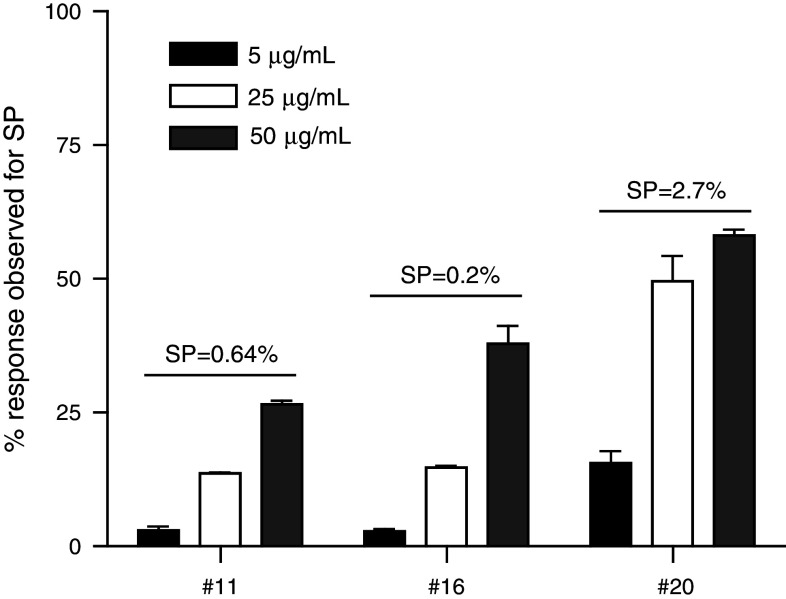
High SLP concentration is required to measure CD8+ T-cell responses directly ex vivo*.* Donor monocytes were loaded with 5, 25 or 50 μg/mL SLP and used to stimulate autologous PBMC. The IFN-γ+ CD8+ T-cell responses to these varied concentrations were analyzed. Relative percentages as compared with the response against the SP of IFN-γ+ CD8+ T cells detected after stimulation with SLP are shown for three donors [medium background ± SD in *brackets*, #11 (0.026 ± 0.006), 16 (0.010 ± 0.006) and 20 (0.025 ± 0.002)]. Numbers depicted on *top of the bars* represent the absolute percentage of IFN-γ+ CD8+ T cells detected after stimulation with SP-pulsed monocytes. Responses were measured in triplicate. The intra-assay variation (%CV) is below 30%

We then used this adapted protocol to measure influenza-specific CD8+ T-cell reactivity in a direct ex vivo setting. In order to minimize the risk that CD8+ T cells responding to other epitopes may influence our tests, we compared the response to SP to that of T cells stimulated with a single 30-mer long peptide containing this CD8+ T-cell epitope (SLP). Pilot experiments revealed that even in the presence of IFN-α, the results obtained with SLP-pulsed monocytes were not comparable to that observed after stimulation of T cells ex vivo with SP-pulsed monocytes (not shown), suggesting that under conditions where T cells had not been previously activated and more sensitive to react, the adapted protocol was not useful. Reasoning that the amount of peptide presented at the cell surface of the APC may still form a rate-limiting factor, which cannot be fully compensated by the use of IFN-α, we performed a dose titration curve for the SLP. Monocytes from 3 different HLA-A*0201-positive subjects were loaded with either 5 μg/mL of the SP or an increasing dose (5, 25 and 50 μg/mL) of the SLP along with GM-CSF. The following day, the peptides were removed, and the responder cells (stored away overnight in the incubator) and IFN-α were added for 16–20 h. In all cases, the response to 5 μg/mL SLP-pulsed APC was very low, when compared with the response to SP (Fig. 3). However, in all three cases, the ex vivo response increased when more of the peptide was fed to the monocytes with the best response at 50 μg/mL (Fig. 3). Increasing the dose to 100 μg/mL did not result in better reactivity (not shown). As we had previously observed that poly(I:C) may stimulate the IFN-γ production of T cells [28], the capacity of this compound to increase the sensitivity of the assay using SLP-pulsed monocytes was tested. Analysis of the response of PBMC from two different healthy donors showed a slight increase in the percentage of responding cells up to 88 % of the response detected with SP-pulsed monocytes when compared with monocytes not stimulated with poly(I:C) (online resource 3), suggesting that the addition of poly(I:C) was another useful improvement to the final protocol (online resource 1).

### Responses observed with the adapted protocol for SLP are comparable to that detected with SP tested according to the original protocol

**Fig. 4:**
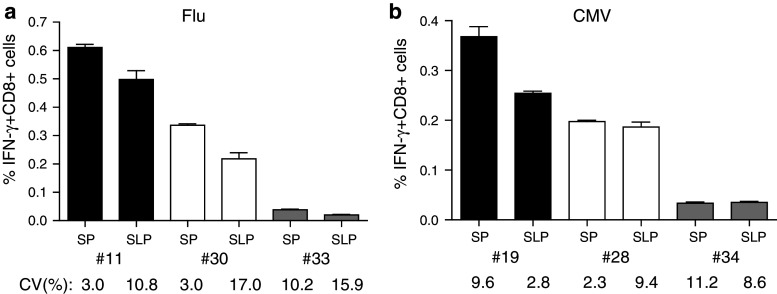
Comparison of CD8+ T-cell reactivities against high SLP and low SP concentration. Monocytes were pulsed with either 5 μg/mL SP or 50 μg/mL single long peptide (SLP) from Influenza M1 (**a**) and human Cytomegalus virus (CMV; **b**). The percentages of IFN-γ+ CD8+ T cells for three donors (Flu #11 (0.002 ± 0.001), 30 (0.007 ± 0.003) and 33 (0.026 ± 0.004); CMV #19 (0.006 ± 0.003), 28 (0.002 ± 0.002) and 34 (0.017 ± 0.005), medium background ± SD) per antigen type are shown. Responses were measured in triplicate. Intra-assay variation (%CV) is shown below the *bars* and was below 30 %

This final adapted protocol for the SLP (50 μg/mL peptide, poly(I:C), IFN-α) then was compared with the original protocol for the SP in another set of 3 HLA-A*0201+ subjects displaying different frequencies of influenza-specific IFN-γ-producing CD8+ T cells. The percentage of IFN-γ+ CD8+ T cells varied between 0.04 and 0.6 % when the SP was used, and this was comparable to the response detected with the adapted protocol for the use of SLP (0.03–0.5 %; Fig. 4a). Depending on the donor, the frequencies of the response against SLP were between 50 and 80 % of that detected with the SP. To confirm these data, similar experiments were performed to compare the response to the HLA-A*0201-restricted CMV-derived CD8+ T-cell epitope. Three donors with high-, intermediate- and low-frequencies of CMV-specific IFN-γ+ CD8+ T cells were used. Again, the CD8 responses detected when the CMV SLP was used ranged between 60 and 95 % of that detected with the CMV SP (Fig. 4b). The intra-assay variation in these experiments was low and varied between 2 and 16 %. In conclusion, the adapted ICS protocol for the use of long peptides to measure CD8+ T-cell responses directly ex vivo is robust and is able to measure at least half of the reactivity that can be found when the exact minimal peptide is known and can be used to stimulate T cells.

### Both CD8+ and CD4+ T-cell responses are directly ex vivo detected in one single ICS assay

**Fig. 5:**
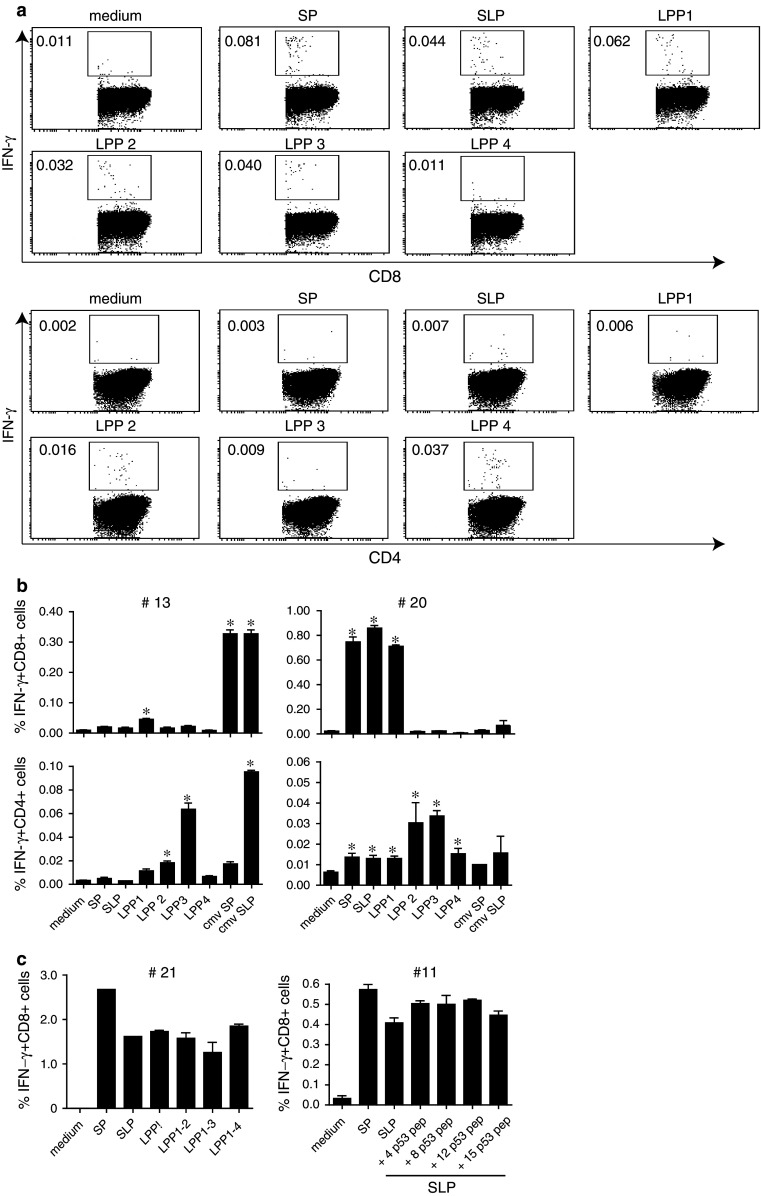
Ex vivo detection of CD4+ and CD8+ IFN-γ-responses in cryopreserved PBMC. PBMC stimulated with SP-, SLP- or LPP-pulsed monocytes were stained for CD4, CD8 and IFN-γ and analyzed by flow cytometry. The flow cytometric plots from the analysis of the response measured in a representative donor (#12) are shown in **a**. The percentages of the measured CD4+ (*lower part*) and CD8+ (*upper part*) T-cell responses are indicated for each condition. Samples were analyzed using the gating strategy as mentioned in online resource 1. **b** Simultaneous measurement of IFN-γ+ CD8+ T-cell responses (*upper row*) and IFN-γ+ CD4 T-cell responses (*lower row*) in cryopreserved PBMC samples of different donors (#13, 20) are shown. The responses are measured in triplicate. The *asterisks* represent positive response (two times higher than the non-stimulated cells, i.e., medium control). **c** Addition of either relevant influenza M1-derived LPPs to SLP (#21) or varying number of irrelevant peptides (from 4, 8, 12 up to 15 peptides) derived from p53 protein (#11) to influenza M1-derived SLP does not affect the percentage of IFN-γ-producing CD8+ T cells responding to the specific SLP when measured ex vivo in a cryopreserved sample

Most optimally, the adapted assay should allow the measurement of both CD8+ and CD4+ T-cell responses in one run as especially patient material is limited. In order to investigate this, monocytes from HLA-A*0201 donors were loaded overnight with either 5 μg/mL SP, 50 μg/mL SLP or 4 different LPP, each consisting of four peptides at a concentration of 50 μg/mL per peptide and used the next day as stimulator cells for the non-adherent PBMC, which were stored overnight in the incubator. The LPP used here were the same as used in our 4-day T-helper IFN-γ-ELISPOT assays (see M&M section, the 4 LPP span the whole Influenza M1 protein) to measure Influenza-specific T-cell responses as well as to obtain some information about the breadth of the T-cell response [11, 19]. We observed IFN-γ-producing T cells both in the CD8 and CD4 gated population. In order to avoid false positive detection, we applied a strict gating strategy (online resource 2). An example of these plots is shown in Fig. 5a. The CD8+ T cells showed reactivity against the SP as well as the SLP and LPP1, both containing the long peptide comprising the HLA-A*0201 restricted CD8+ T-cell epitope, albeit the response to the SLP was slightly lower. In addition, LPP 2 and 3 were also recognized, whereas LPP4 was not. The CD8+ T-cell responses to these other peptide pools were at the same level as the response against the known HLA-A*0201 epitope. The CD4+ T cells responded to all LPP with a similar response profile as seen in the IFN-γ-ELISPOT, in which also the responses to LPP2 and 4 were the strongest (data not shown). Two other representative examples are shown in Fig. 5b. In one donor (#13), the CD8+ T cells only responded to the CMV SP and SLP, but not to those of influenza. There was a weak CD8+ T-cell response to LPP1. The CD4+ T cell population, however, clearly showed a response to LPP 2 and 3 as well as to the CMV SLP, but not to CMV SP (Fig. 5b). Notably, the CMV SLP contains a pan HLA-DR epitope [29, 30]. The other donor (#20) showed a strong CD8+ T-cell response to SP, SLP and LPP1, and not to the other peptide pools. The CD4+ T-cell reactivity was found against all LPPs, but most dominantly against LPP 2 and 3. The CD8+ and CD4+ T-cell responses from 9 additional donors tested against all these antigens using this adapted ICS protocol are shown in online resource 4. More than 80 % of the responses detected in ICS were similar to the responses detected by ELISPOT (data not shown).

In order to test whether the number of peptides used in the LPP would influence the percentage of cells recognizing a particular epitope, we tested the PBMC from one donor against the SP, SLP and different LPPs comprising 4, 8, 12 or 16 of the Influenza long peptides. In another case, the influenza SLP was mixed with 4, 8, 12 or 15 different p53 SLP [31] to form LPPs of different sizes. In both cases, the response to the SLP was not altered by the addition of extra “irrelevant” peptides (Fig. 5c).

All together, our results show that the adapted ICS protocol allows the ex vivo detection of low-frequency antigen-specific CD4+ and CD8+ T cells to both known and unknown epitopes. Importantly, the reaction is not altered by the addition of extra peptides, which indicates that the antigen-specific reactions in PBMC samples can be screened by using pools of peptides and can, therefore, be very useful in the immunomonitoring of vaccination trials.

### The adapted ICS protocol is superior in detecting low-frequency p53-specific CD4+ T-cell responses in PBMC from patients participating in a p53 vaccine trial

**Fig. 6:**
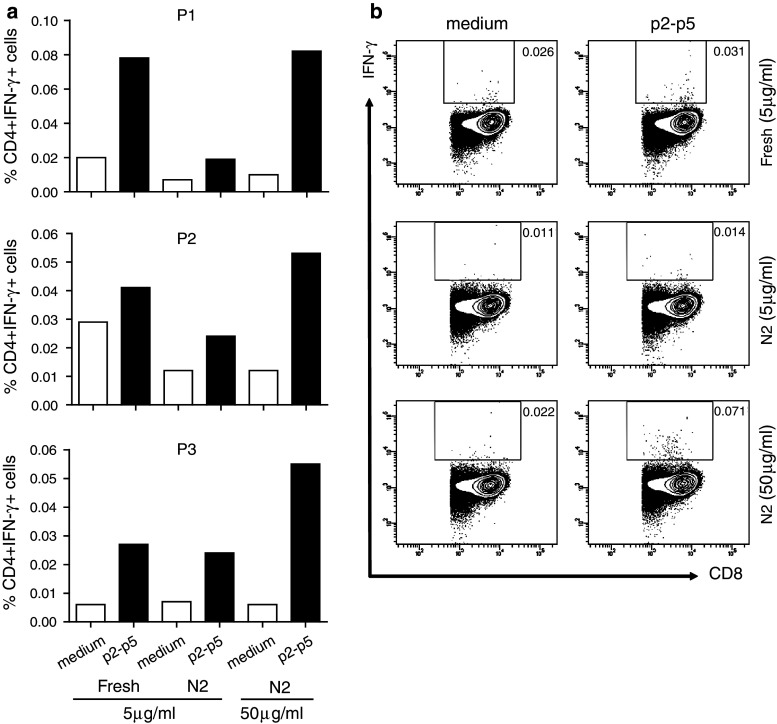
The adapted ICS protocol also improves the detection of antigen-specific CD4+ T cells in cryopreserved samples of patients participating in a p53 vaccine trial. Post–vaccination, PBMC samples were analyzed freshly by the old protocol using a low peptide concentration of 5 μg/mL. Non-stimulated PBMC (medium) are taken along as negative control. In addition, the cryopreserved PBMC of these patients were simultaneously analyzed by using either 5 or 50 μg peptide per mL. The percentages of CD4+ IFN-γ+ T cells in **a** and CD8+ IFN-γ+ T cells in (P3 only) **b** are depicted

We had the opportunity to test the adapted protocol with a few PBMC samples of an ongoing clinical trial in which patients with colorectal cancer were injected with a p53 vaccine. In this trial, patient’s PBMC were tested freshly ex vivo using the non-adjusted protocol as only CD4+ T-cell reactivity was expected [31]. PBMC of each patient was tested against medium (negative control) and a p53 LPP, consisting of all long peptides of the p53 vaccine, and revealed that in all three cases, IFN-γ-producing CD4+ T cells were present (Fig. 6a). After freeze-thawing, the same patient’s PBMC were tested again using the non-adjusted protocol (N2, 5 μg/mL per peptide) and by applying the adapted protocol (N2, 50 μg/mL per peptide). Clearly, the response of thawed PBMC was lower than that of freshly isolated and tested PBMC when the non-adjusted protocol was used. In contrast, the adapted protocol restored the response of the CD4+ T cells to a similar or slightly higher level as previously detected in the freshly tested PBMC samples (Fig. 6a). While the CD8 T-cell response to p53 is known to be blunted, this is not complete, and p53-specific CD8 T-cell responses can be induced by vaccination. Indeed, in one of the patients (P3), a p53-specific CD8 T-cell response was detected by the optimized protocol, but not the original protocol (Fig. 6b). Overall our data show that a high concentration of peptide is required for the optimal processing and presentation of T-cell epitopes in both HLA class I and II to allow the ex vivo detection of antigen-specific CD4+ and CD8+ T cells present in cryopreserved PBMC.

## Discussion

In this study, we have improved an ICS protocol to directly ex vivo detect low to high frequencies of antigen-specific CD4+ and CD8+ T-cell responses, by using pools of long peptides. Previously, we had shown that SLP and LPP could perfectly stimulate antigen-specific CD4+ and CD8+ T cells as well as could be used to detect T-cell reactivity in activated T-cell cultures by ICS or other assays that lasted a couple of days [10, 11, 16], but the existing ICS protocol failed to detect T-cell reactivity directly ex vivo in cryopreserved samples. The ability to directly ex vivo measure low-frequency CD8+ and CD4+ T-cell responses using SLP or LPP depended on the concentration of the long peptides used, which needed to be at least tenfold higher, and was enhanced by compounds that increased processing and presentation of these peptides by the APC, in our hands IFN-α and poly(I:C). Furthermore, our data indicated that the presence of other peptides (up to 16 different long peptides)—which theoretically may congest the uptake and processing machinery—did not alter the processing and presentation of SLP. All together, this suggests that especially the capacity to ingest and process enough amounts of peptide within the time frame used here is the rate-limiting factor for sufficient presentation of peptide epitopes in HLA class I and II. This is not a factor when exact HLA-fitting peptide epitopes are used as they can bind to the HLA molecules at the cell surface without a requirement for uptake and processing. Also for the mouse OVA model, it has been shown that a higher protein concentration resulted in a more efficient cross-presentation to antigen-specific CD8+ T cells [32, 33].

In addition to an enhanced cross-priming [25], IFN-α also upregulates the co-stimulatory molecule CD80 on monocytes, a molecule essential for T-cell activation [34, 35]. Poly(I:C) is also known to activate and enhance the expression of co-stimulatory molecules on APC [36, 37]. Improved co-stimulation may explain part of the increased reactivity to SLP-/LPP-pulsed monocytes. Recent studies in the field of HIV have shown the use of antibodies to CD28 and CD49d to co-stimulate T cells during in vitro stimulation to enhance their reactivity [38, 39]. Possibly, the use of such antibodies in our protocol may enhance responsiveness also, but this was not tried as also an increase in background reactivity was observed in the aforementioned studies [39], and this is known to affect the capacity to detect low-frequency responses [18, 24, 40]. Finally, IFN-α is known to enhance the survival of activated T cells [41], and one can envisage that keeping the activated cells alive during the test increases the detection efficiency of low-frequency T cells.

While comparing the responses against the exact CD8+ peptide epitope (SP) with SLP or LPP of either influenza M1 or CMV, we observed that CD8+ T-cell responses had a reactivity toward a SLP ranging between 50 and 90 % of the response detected by applying the SP as antigen. In situations where the T-cell epitopes are known and patients are included based on the restricting HLA types, it is, therefore, preferred to use the exact HLA-fitting peptides for stimulation of the PBMC. In other cases, for instance when vaccines are used that encode for whole proteins or comprise whole proteins and the patient population constitute a multitude of different HLA types, LPPs may be used to detect a number of CD4+ and CD8+ T-cell responses within one or more patients. This can be important as we have recently shown that both the breadth and the magnitude of a T-cell response was associated with the clinical response to vaccination [8, 10], however, with the trade-off that some responses can be missed.

Applying our previous and adapted protocol to PBMC samples of patients vaccinated in an ongoing p53-vaccination trial revealed that the new protocol also displayed better performance with respect to the detection of CD4+ and CD8+ T-cell responses. The responses measured in cryopreserved PBMC by the old protocol were clearly inferior to those measured when freshly isolated. The adapted protocol, however, restored the measured activity to the levels found with freshly isolated PBMC.

In conclusion, here we present a successfully improved and robust ICS protocol to detect antigen-specific CD4+ and CD8+ T cells directly ex vivo in cryopreserved PBMC samples using one single vial of PBMC. The assay, thus, is highly economical with respect to the usage of the restricted amounts of patient material generally available for immunomonitoring of cancer trials. Whereas other sensitive and quantitative assays such as the ELISPOT assay also allow for ex vivo measurements, it is important to obtain as much information about the reacting T cell as possible since trial samples are unique. The current assay allows for more detailed analysis of the T-cell phenotype as it utilizes flow cytometry. The costs for the assay are higher, but assay costs are less important when one considers the expenditure of a trial. With this protocol, we have consistently measured antigen-specific responses with intra- and inter-assay variation below 30 % as well as were able to detect low-frequency responses. This makes the improved assay suitable for the monitoring of samples from anticancer vaccination trials.

### Electronic supplementary material

Below is the link to the electronic supplementary material.

Supplementary material 1 (PDF 3.32 mb)
