# Supplementary material for: The simultaneous ex vivo detection of low-frequency antigen-specific CD4+ and CD8+ T-cell responses using overlapping peptide pools
Source: Cancer Immunol Immunother. 2012 Apr 11;61(11):1953–63. doi: 10.1007/s00262-012-1251-3 (PMC3493661; doi:10.1007/s00262-012-1251-3)
Supplement: Supplementary file 1 — Supplementary material 1 (PDF 3.32 mb) [file 262_2012_1251_MOESM1_ESM.pdf]

The simultaneous *ex-vivo* detection of low frequency antigen-specific CD4+ and CD8+ T-cell responses using overlapping peptide pools.

Satwinder Kaur Singh[1], Maaike Meyering[1], Tamara H. Ramwadhdoebe[1], Linda F.M. Stynenbosch[1],Anke Redeker[2], Peter J.K. Kuppen[3], Cornelis J.M. Melief[2], Marij J.P. Welters[1] and Sjoerd H. van der Burg[1]

Departments of [1]Clinical Oncology, [2]Immunohematology and Blood Transfusion and [3]Surgery, Leiden University Medical Center, Leiden, The Netherlands.

Corresponding author S.H. van der Burg, email: shvdburg@lumc.nl

Online resource 1: Final ICS protocol

Standard Operating Procedure: Indirect analysis of low frequency antigen-specific T-cells by multiparameter flow cytometry.

Materials:

Brefeldin A (5mg/ml in absolute ethanol)	Sigma (cat no. B-7651) according to appendix 08
Phosphate Buffered Saline (PBS)	Fresenius Kabi
PBS / 0.5% BSA/0,02% NaAz	according to appendix 02 *(see note below)*
4% Paraformaldehyde in PBS	according to appendix 03 not older than 1 month!
1% Paraformaldehyde in PBS	Delivered by LUMC
10 % saponine in PBS	Sigma (cat no. S7900) according to appendix 04

PBS / 0.5%BSA / **0.1% Saponin** / NaAz 0.02%			*(see note below)*
PBS / 0.5%BSA / **10% FCS** / 0.1% Saponin / NaAz 0.02% (freshly made)			*(see note below)*
X-vivo 15 medium			Lonza (cat no. 04-418Q)
IMDM +			according to appendix 01
Fetal calf serum			PAA (cat no. A15-101)
Human AB serum (ABS)			Greiner or equivalent
Antibody from BD	IFN-y	FITC	Cat no. 554551
Antibody from BD	IL-2	PE	Cat no. 554566
Antibody from BD	CD4	PE Cy7	Cat no. 557852
Antibody from BD	CD8	APC Cy7	Cat no. 348813
Antibody from BD	CD137	APC	Cat no. 550890

Antibody from BD	CD154 PE Cy5	Cat no. 555701
Antibody from DAKO	CD3 Pacific Blue	Cat no. PB982
Tubes (15 ml and 50 ml)		
24 wells plate		
Peptides		
96 wells plate V-bottom		Greiner (Cat no. 651101)
Roferon-A=IFN-alpha 2a ($3*10^6$ IU/0,5ml)		Cat no. 972312 delivered by LUMC
recombinant human GM-CSF (800000 U/ml)		Biosource (Cat no. PH C2011)
Poly(I:C) (2.5 mg/ml or 1 mg/ml)		Invivogen (Cat no. tlrl-pic-5)

Note: optionally no NaAz is added to the PBS / 0.5%BSA/… *solutions, but then it is of outmost importance to keep the stock solutions sterile. For each experiment take the required volume of the solution under sterile conditions (in the flow cabinet) and use it on the bench during the staining whereafter this unsterile solution is thrown away.*

Protocol:

Day 1: Preparation of PBMC for test.

- Thaw PBMC according to protocol LUMC-ECIT 011-10.
- Make a concentration of min. $1*10^6$ cells/ml and max. $4*10^6$ cells/ml in X-vivo 15 medium.
- Add 1 ml of PBMC to each well of a 24-well plate.
- Allow the monocytes to adhere for 2 hours at 37°C and 5% CO_2 (92% humidity).
- Just prior to removal of non-adherent cells prepare X-vivo 15 medium with 800 U/ml GM-CSF (*Note that this solution has to be made freshly*).
- Transfer non-adherent cells into a 50 ml tube.
- Add 1 ml of plain IMDM to monocytes.
- Gently resuspend IMDM to wash the monocytes.
- Transfer the remaining non-adherent cells also to the 50 ml tube.
- Add 1 ml X-vivo 15 medium + 800 U/ml GM-CSF per well to the monocytes.
- Also add peptide pools to the X-vivo 15 medium on the monocytes according to the following final concentration:

Medium alone as negative control	
Short peptides	5µg/ml
Long peptides or peptide pools	50 µg/ml
And later PHA as positive control	0.5µg/ml

  (*Note that PHA is only added to responder cells and not to monocytes)*
  (Note that not more than 16 peptides should be used for stimulation in 1 sample)
  (Note that stock concentrations of peptides should not be higher than 1 mg/ml, since it may contain max. 8% DMSO in PBS, due to higher mortality and lower responses of your cells if this is exceeded)
- Culture monocytes for 5 hours at 37°C and 5% CO_2 (90-92% humidity).
- Spin non-adherent cells in 50 ml tube for 5 minutes at 1600 rpm.
- Resuspend non-adherent cells in a 10 ml of IMDM + 10% ABS.

- Count the non-adherent cells (number of viable and death cells using trypan blue staining).
- Store non-adherent cells in 50 ml tube with cap slightly open at 37°C and 5% CO_2 (90-92% humidity) (*note that max. $2*10^6$ cell/ml and max. 10 ml per tube can be stored*).
- 5 hours after peptide loading of the monocytes, add poly(I:C) to a final concentration per well of 25 µg/ml.
- Culture monocytes overnight at 37°C and 5% CO_2 (90-92% humidity).

Day 2: Activation of Responder cells.

- Spin non-adherent cells (further mentioned as responder cells) for 5 minutes at 1600 rpm.
- Resuspend responder cells in desired volume IMDM + 10% ABS, depending on the number of samples (1 ml per sample).
- Count the responder cells and make a concentration of max. $2*10^6$cells/ml in IMDM + 10% ABS.
- Gently resuspend the medium of the cultured peptide-loaded monocytes.
- Remove the medium from the monocytes.
- Add 1 ml of responder cells per well.
- Then add 16.7 µl/well of Roferon-A (final concentration is 10^5 IU/ml) to each well.
- Incubate the cell cultures for 1 hour at 37°C and 5% CO_2 (90-92% humidity).
- Make a solution of 110 µg/ml Brefeldin A in IMDM + 10% ABS (freshly made from stock solution of Brefeldin A of 5 mg/ml).
- Add 100µl/well of 110µg/ml Brefeldin A (final concentration is 10µg/ml) to the cultures.
- Incubate overnight at 37°C and 5% CO_2 (90-92% humidity).

Day 3: Staining.

- Harvest the cells in the 24-wells plate and collect them in a 15 ml tube. (*Note: resuspend the cells carefully so that the monocytes will remain adhered to the bottom of the plate).*
- Spin the tubes for 5 minutes at 1600 rpm at 6 degrees Celsius.
- Remove the supernatant carefully.
- Resuspend the cells in 100 µl of cold (4°C) PBS / 0.5% BSA/0.02% NaAz.
- Transfer these cells into V-bottom 96-wells plate (*Note: maximally $1*10^6$ cells/well, otherwise divide the cells over 2 or more wells)*
- Wash twice with 100 µl of cold (4°C) PBS / 0.5% BSA (spin the tubes for 2 minutes at 1400 rpm at 6 degrees Celsius) and remove supernatant.
- Fixate cells in 50µl of 4% Paraformaldehyde for 4 minutes on ice.
- Add 100 µl cold PBS / 0,5% BSA/0,02% NaAz.

- Spin the cells in the V-bottom plate for 2 minutes at 1400 rpm at 6 degrees Celsius.
- Remove the supernatant.
- Wash cells again with 100 µl of cold PBS / 0.5% BSA/0,02% NaAz.
- Wash cells with 100µl of cold PBS / 0.5%BSA / **0.1% Saponin** / NaAz 0.02%.
- Incubate cells with 50µl of PBS / 0.5%BSA / **10% FCS** / 0.1% Saponin / NaAz 0.02% for 10 minutes on ice.
- Add cold 100 µl of PBS / 0.5%BSA / **0.1% Saponin** / NaAz 0.02%, spin down and remove supernatant.
- Add 50 µl of the following antibody solution:

37.5 µl PBS / 0.5%BSA / **0.1% Saponin** / NaAz 0.02%

1µl	IFNγ-FITC	(1:50 diluted)
1µl	IL2 – PE	(1:50 diluted)
1µl	CD4-PE Cy7	(1:50 diluted)
1µl	CD8-APC Cy7	(1:50 diluted)
2.5µl	CD3 Pacific Blue	(1:20 diluted)
1µl	CD137 APC	(1:50 diluted)
5µl	CD154 PECy5	(1:10 diluted)

- Resuspend the cells gently in this antibody solution.
- Incubate for 30 minutes in the dark on ice.
- Add 100 µl of PBS / 0.5%BSA / **0.1% Saponin** / NaAz 0.02%, spin down and remove supernatant.
- Wash once more with PBS / 0.5%BSA / **0.1% Saponin** / NaAz 0.02%.
- Resuspend in 100µl/well of 1% paraformaldehyde.
- Transfer these fixed and stained cells into flow cytometric tubes.
- Store the samples until acquisition by flow cytometry in the dark at 4°C.
  (Note: Store samples maximally for 24 hours before measurement)

Prior to acquisition create compensation controls

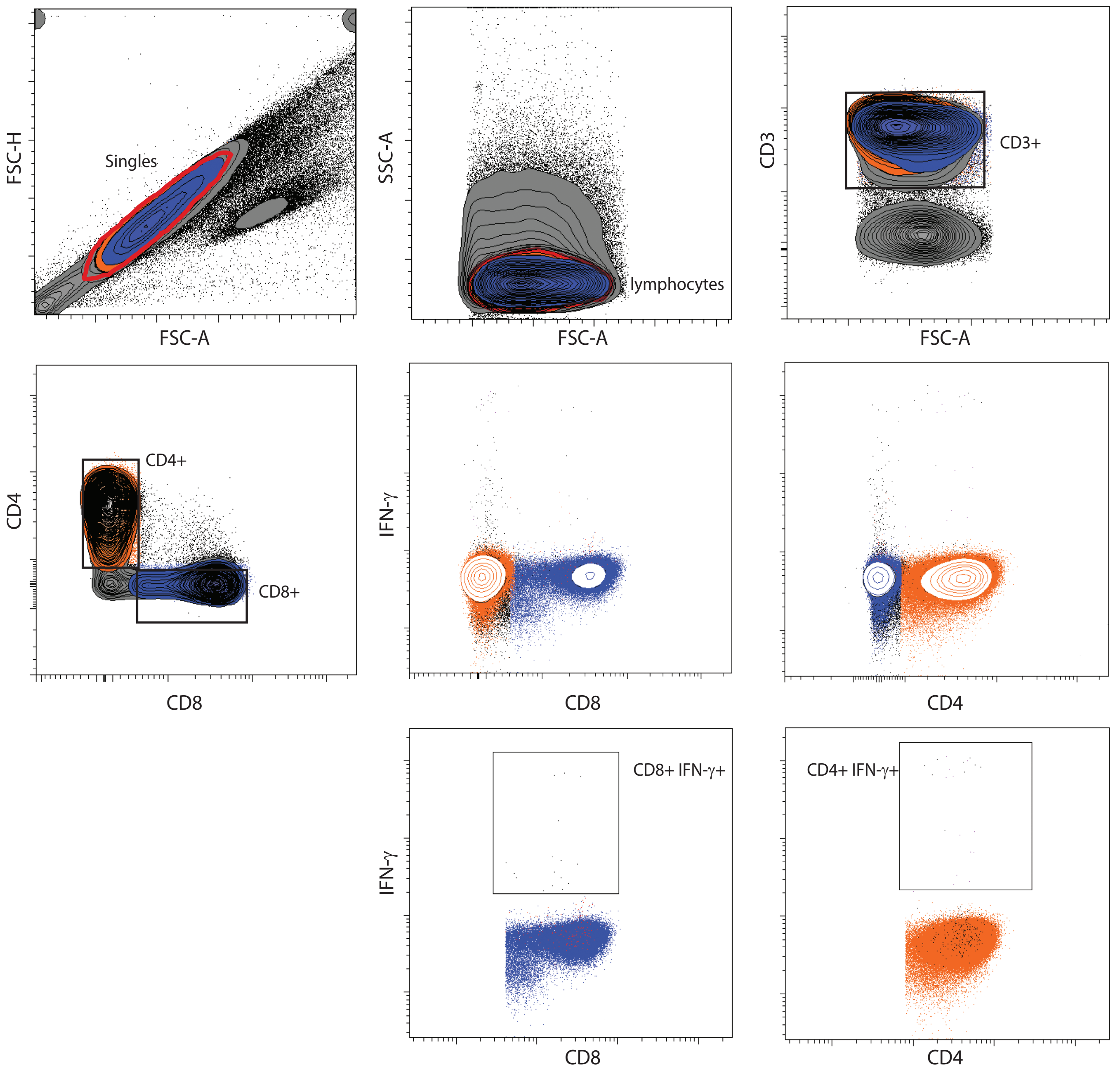

The simultaneous *ex-vivo* detection of low frequency antigen-specific CD4+ and CD8+ T-cell responses using overlapping peptide pools.

Satwinder Kaur Singh[1], Maaike Meyering[1], Tamara H Ramwadhdoebe[1], Linda F.M. Stynbosch[1], Anke Redeker[2], Peter J.K. Kuppen[3], Cornelis J.M. Melief[2], Marij J.P. Welters[1] and Sjoerd H. van der Burg[1]

Department of [1]Clinical Oncology, [2]Immunohematology and Blood Transfusion and [3]Surgery. Leiden University Medical Center, Leiden, The Netherlands.

Corresponding author: S.H van der Burg, email: shvdburg@lumc.nl

Online resource 2: Gating strategy applied to analyze antigen-specific CD8+ and CD4+ T cells directly ex-vivo.
In order to avoid false positive detection we applied a strict gating strategy. The single cells were first gated excluding the doublets, which can lead to higher frequencies of positive cells. Next, the lymphocytes were gated in this single cell population. The lymphocytes were further gated on CD3+ T cells, which further were divided on the basis of CD8 and CD4 expression. The gates were applied on dense CD8 and CD4 populations; both high and intermediate CD8 or CD4 expressing cells were included as these cells can actively produce IFN-γ. These gated CD4+ and CD8+ T cells were further analysed and plotted either as CD8 or CD4 against IFN-γ. The IFN-γ was set higher to prevent high ~~backgroung~~. Using this gating strategy we were able to measure the frequency of both CD8+ and CD4+ T cells that produce IFN-γ upon specific antigen recognition.

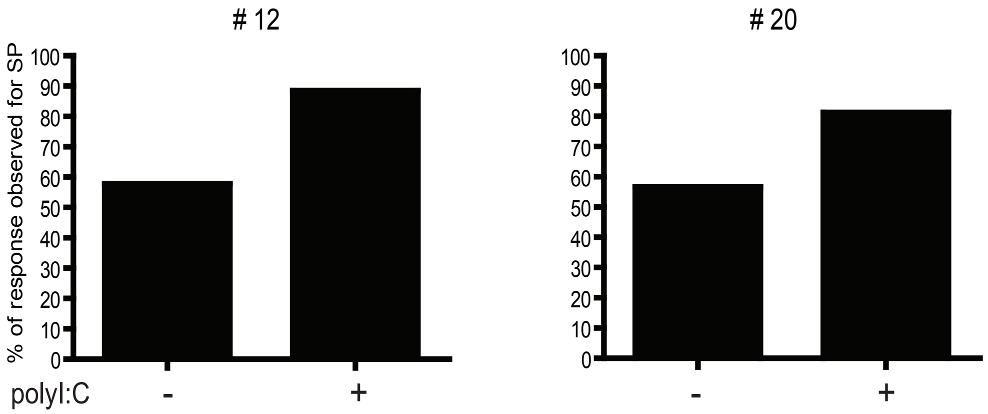

The simultaneous *ex-vivo* detection of low frequency antigen-specific CD4+ and CD8+ T-cell responses using overlapping peptide pools.

Satwinder Kaur Singh[1], Maaike Meyering[1], Tamara H Ramwadhdoebe[1], Linda F.M. Stynbosch[1], Anke Redeker[2], Peter J.K. Kuppen[3], Cornelis J.M. Melief[2], Marij J.P. Welters[1] and Sjoerd H. van der Burg[1]

Department of [1]Clinical Oncology, [2]Immunohematology and Blood Transfusion and [3]Surgery. Leiden University Medical Center, Leiden, The Netherlands.

Corresponding author: S.H van der Burg, email: shvdburg@lumc.nl

Online resource 3: Poly(I:C) boost antigen-specific T-cell responses
Monocytes were pulsed either with SP or SLP derived from influenza M1 and subsequently stimulated with or without TLR3-ligand poly(I:C) overnight. Relative percentages as compared to the response observed after with SP of IFN-γ+CD8+ T cells detected after stimulation with SLP in the ~~absernce~~ or presence of poly(I:C) is depicted for two different donors (#12, 20)

donors		% CD8	medium	flu SP	flu SLP	flu LPP1	flu LPP2	flu LPP3	flu LPP4	cmv SP	cmv SLP
#10	mean	27,74	0,006	**0,333**	**0,227**	**0,281**	**0,032**	0,009	0,007	**0,024**	**0,023**
	SD		0,002	0,016	0,017	0,006	0,006	0,003	0,003	0,003	0,004
#19	mean	32,11	0,006	**0,020**	0,008	**0,044**	**0,022**	0,011	0,007	**0,354**	**0,320**
	SD		0,003	0,005	0,003	0,007	0,001	0,003	0,001	0,006	0,009
#22	mean	18,31	0,012	**0,330**	**0,263**	**0,293**	0,012	0,015	0,010	0,012	0,011
	SD		0,003	0,014	0,014	0,007	0,005	0,002	0,003	0,006	0,003
#23	mean	22,67	0,026	**0,085**	**0,049**	**0,078**	0,036	0,020	0,013	**1,076**	**1,111**
	SD		0,008	0,010	0,014	0,013	0,002	0,007	0,002	0,041	0,085
#25	mean	39,16	0,004	**0,015**	0,007	**0,027**	0,005	0,006	0,005	**0,031**	**0,027**
	SD		0,001	0,002	0,001	0,003	0,002	0,001	0,002	0,002	0,003
#28	mean	31,41	0,002	**0,025**	**0,016**	**0,024**	**0,010**	**0,004**	**0,008**	**0,147**	**0,149**
	SD		0,002	0,001	0,003	0,006	0,003	0,003	0,001	0,013	0,017
#30	mean	23,33	0,012	**0,156**	**0,154**	**0,165**	**0,030**	**0,035**	0,020	**0,054**	**0,027**
	SD		0,008	0,030	0,017	0,027	0,002	0,006	0,006	0,041	0,012
#33	mean	27,65	0,007	**0,054**	**0,039**	**0,038**	0,012	0,005	0,008	0,006	0,007
	SD		0,002	0,004	0,001	0,007	0,001	0,002	0,006	0,006	0,002
#34	mean	27,46	0,015	**0,092**	**0,063**	**0,066**	0,024	**0,036**	**0,037**	**0,054**	**0,049**
	SD		0,007	0,008	0,005	0,001	0,006	0,009	0,004	0,004	0,004

donors		% CD4	medium	flu SP	flu SLP	flu LPP1	flu LPP2	flu LPP3	flu LPP4	cmv SP	cmv SLP
#10	mean	65,14	0,003	0,004	0,004	**0,010**	0,005	**0,007**	**0,010**	0,004	**0,009**
	SD		0,001	0,002	0,001	0,001	0,001	0,004	0,001	0,002	0,004
#19	mean	61,74	0,003	0,004	0,003	0,005	**0,008**	**0,007**	**0,009**	0,005	0,004
	SD		0,001	0,002	0,001	0,002	0,001	0,000	0,002	0,000	0,002
#22	mean	76,87	0,007	0,008	0,012	0,008	0,013	**0,014**	0,008	0,012	0,008
	SD		0,002	0,003	0,002	0,001	0,001	0,003	0,003	0,006	0,002
#23	mean	69,83	0,002	0,003	0,003	**0,006**	**0,004**	**0,014**	**0,011**	**0,004**	**0,011**
	SD		0,001	0,001	0,001	0,002	0,001	0,004	0,002	0,000	0,002
#25	mean	52,33	0,002	**0,004**	0,003	**0,004**	**0,017**	**0,018**	**0,013**	**0,004**	**0,008**
	SD		0,001	0,001	0,001	0,000	0,001	0,005	0,003	0,001	0,002
#28	mean	64,07	0,003	**0,006**	**0,006**	**0,013**	**0,010**	0,004	**0,020**	0,005	0,004
	SD		0,001	0,001	0,001	0,004	0,001	0,002	0,001	0,001	0,001
#30	mean	70,04	0,005	0,007	**0,012**	**0,012**	**0,020**	**0,023**	**0,013**	0,008	**0,012**
	SD		0,001	0,002	0,001	0,002	0,001	0,006	0,002	0,002	0,003
#33	mean	61,46	0,003	0,004	**0,006**	**0,009**	**0,015**	**0,006**	**0,007**	**0,006**	0,003
	SD		0,001	0,001	0,001	0,002	0,002	0,001	0,001	0,002	0,001
#34	mean	67,28	0,009	**0,018**	0,016	**0,018**	0,015	0,017	**0,021**	0,016	0,017
	SD		0,002	0,003	0,008	0,004	0,001	0,002	0,005	0,005	0,004

The simultaneous *ex-vivo* detection of low frequency antigen-specific CD4+ and CD8+ T-cell responses using overlapping peptide pools.

Satwinder Kaur Singh[1], Maaike Meyering[1], Tamara H Ramwadhdoebe[1], Linda F. M. Stynbosch[1], Anke Redeker[2], Peter J.K. Kuppen[3], Cornelis J.M. Melief[2], Marij J.P. Welters[1] and Sjoerd H. van der Burg[1]

Department of [1]Clinical Oncology, [2]Immunohematology and Blood Transfusion and [3]Surgery.
Leiden University Medical Center, Leiden, The Netherlands.

Corresponding author: S.H van der Burg, email: shvdburg@lumc.nl

Online resource 4: *Ex-vivo* detection of influenza-specific IFN-γ-producing CD4+ and CD8+ T cells in a group of healthy donors.
The percentages of CD8+ (top table) and CD4+ (lower table) T cells producing IFN-γ when stimulated with indicated influenza M1- or CMV-derived SP, SLP and LPP detected in 9 different donors.
Responses shown are the mean of triplicates. Bold numbers represent positive responses detected for either influenza M1 or CMV peptides in different donors.
